# First Record of the Alien Species *Procambarus virginalis* Lyko, 2017 in Fresh Waters of Sardinia and Insight into Its Genetic Variability

**DOI:** 10.3390/life11070606

**Published:** 2021-06-24

**Authors:** Daria Sanna, Ilenia Azzena, Fabio Scarpa, Piero Cossu, Angela Pira, Flavio Gagliardi, Marco Casu

**Affiliations:** 1 Dipartimento di Scienze Biomediche, Università di Sassari, Viale San Pietro 43/B, 07100 Sassari, Italy; iazzena@uniss.it; 2 Dipartimento di Medicina Veterinaria, Università di Sassari, Via Vienna 2, 07100 Sassari, Italy; fscarpa@uniss.it (F.S.); picossu@uniss.it (P.C.); marcasu@uniss.it (M.C.); 3 Acquario di Cala Gonone, Via La Favorita, 08020 Cala Gonone, Italy; acquariologia@acquariocalagonone.it (A.P.); flaviogagliardi@panaque.com (F.G.)

**Keywords:** alien species, invasive species, non-indigenous crayfish, biological invasion, marbled crayfish, Sardinian fresh waters, mtDNA

## Abstract

In the fresh waters of Sardinia (Italy), the non-indigenous crayfish species *Procambarus clarkii* has been reported from 2005, but, starting from 2019, there have been several reports of a new non-indigenous crayfish in southern and central areas of this Mediterranean island, and its morphology suggests that this species may be the marbled crayfish *Procambarus virginalis*. Forty-seven individuals of this putative species were analyzed, using the mitochondrial gene Cytochrome c Oxidase subunit I as molecular marker to identify this crayfish and investigate the level of genetic variability within the recently established population. Phylogenetic and phylogeographic analyses were carried out on a dataset including sequences from the Sardinian individuals and from all congenerics available in GenBank. Results showed that the new Sardinian crayfish belong to the species *P. virginalis*. All the sequences belonging to *P. virginalis* from European countries are identical, with only few exceptions found among Sardinian individuals. In conclusion, this paper highlights the occurrence of a new further alien species in the Sardinian fresh waters, which are already characterized by the high presence of non-indigenous species.

## 1. Introduction

The so-called freshwater crayfish (Malacostraca, Decapoda) are a monophyletic group of crustaceans with challenging taxonomy and phylogeny [1,2,3], which are present in each continent, except for Antarctica and mainland Africa [4,5], and occur in almost every type of freshwater habitat, both lentic and lotic [5,6,7].

As they are among the largest invertebrate predators in their habitats, freshwater crayfish are an important component in the structuring of the aquatic fauna [8,9]. Indeed, many studies showed a clear impact as a keystone species, due to their feeding activity, which mainly involves vegetation, invertebrates, and vertebrates such as fish. Furthermore, as ecosystem engineers, they can create major impacts on their habitat, affecting the sediment transport as a consequence of their constant search for new refugia, from riffles to pools and vice versa [7,8].

Over the years, multiple crayfish species were translocated, either deliberately or accidentally [10,11,12]. For this reason, at least one non-indigenous crayfish species (hereafter NICS, following Holdich [13]) has been reported in most European countries, and the general number of NICS is rapidly growing [14,15,16].

The presence of NICS can have serious consequences on native ecosystems [17], not only due to interspecific competition with indigenous species or habitat modification, but also by carrying diseases, such as the “crayfish plague”, i.e., *Aphanomyces astaci* [17]. This water mould is mainly transmitted by the largely resistant introduced North American crayfish species, and can lead to high levels of mortality, causing declines of indigenous fauna [18,19,20]. Furthermore, as this oomycete can survive on fish skin and use these animals as vectors, another way of transmission is represented by fish moving away from areas where the crayfish plague is present, which can thus spread the infection among drainage basins [21].

In Europe, there are few native species of crayfish [22,23,24], and the first documented introduction of a NICS dates back to 1890 in Barnowko village (Poland), where the spiny-cheek crayfish, *Faxonius limosus* (Rafinesque, 1817), was introduced for commercial purposes [25] from Pennsylvania (USA). It was followed by other NICS introductions, mainly *Procambarus clarkii* Girard, 1852 (firstly introduced in Spain in 1973), which is considered as a very harmful problematic NICS due to the plastic life cycle, capability of naturalization, and rapid potential for dispersal [26,27].

Later, in the 90s, in a German pet trade, a new species was identified for the first time, it was the marbled crayfish, Procambarus virginalis Lyko, 2017 [28].Preliminary studies hypothesized that *P. virginalis* might represent the product of hybridization between the sexually reproducing *Procambarus fallax* (Hagen, 1870) (from FL, USA) and its congeneric *P. clarkii* (from LA, USA) [29]. However, recent studies have highlighted the autopolyploid nature of the marbled crayfish, which is a triploid organism that differentiated from its mother species *P. fallax* [30,31]. All marbled crayfish show common phenotypic, genetic, and epigenetic characteristics, despite their broad geographical distribution [31] and reproduce by parthenogenesis, being able to quickly create “wild” populations in the temperate zones of the planet [32,33].

Considering the high interest in the marbled crayfish from aquarists, their presence in European ecosystems is probably due to the voluntary release into the wild carried out by some owners and traders [32,33,34,35,36,37]. To date, stable populations of *P. virginalis* in Europe are known from: Germany and Netherlands [10], Croatia [38], Romania [39], Slovakia [40], Estonia [41], Czech Republic [42], Hungary [43], Ukraine [44], and Sweden [45]. The spread of this species is not limited to Europe, in fact its presence has been also reported in Japan [46] and Madagascar [29,47]. Furthermore, although not yet mentioned in scientific publications, several individuals of marbled crayfish have been discovered in different freshwater areas of Poland, Taiwan, and Macau, based on recent local reports. In Italy, (where four autochthonous crayfish species occur [48]), the first report of *P. virginalis* dates back to 2008 [11], but, at present, stable populations are not reported.

In 2019, some individuals morphologically attributable to the marbled crayfish were found in freshwater habitats of southern Sardinia (Italy). Just one year later, individuals that can be identified as marbled crayfish have been found in many other areas of Sardinia (pers. obs.). In this Mediterranean island, two other species of crayfish are known so far: *Austropotamobius pallipes* Lereboullet, 1858, which is indigenous to Italy, and is rarely found in Sardinian freshwaters, where, likely, its occurrence is not natural but due to translocation events [49]; and the NICS *P. clarkii*, which was recorded for the first time in 2005 [50] and it is now widely distributed across the island.

This paper aimed to: (1) identify the new freshwater crayfish found in Sardinia, applying a species delimitation approach; (2) investigate the levels of the species’ genetic variability in Sardinia; and (3) perform phylogenetic and phylogeographic analyses on the newly established population (the only known in Italy), comparing sequences of Sardinian individuals with those already available in the literature, in order to place the data obtained in a wider geographic background.

## 2. Materials and Methods

### 2.1. Sample Collection

Forty-seven individuals were analysed (Table 1), whose morphology was consistent with marbled crayfish or the closely related slough crayfish, *Procambarus fallax* following Hobbs [51].

**Figure 1:**
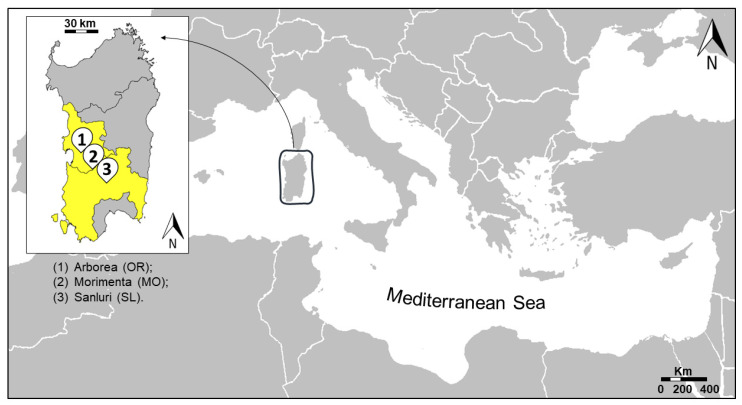
The three geographic areas of the sites where samples were collected are indicated in the map of the Mediterranean island of Sardinia.

Forty-one adult individuals, which were tentatively identified as the marbled crayfish (*Procambarus virginalis*), were collected from May to December 2019 in the central and southern areas of Sardinia which are the alleged center of the first dispersion of this crayfish in the island (see Figure 1 and Table 1 for details). Six eggs (specimens PFMO8–PFMO13 in Table 1), taken from one of the adult individuals, were added to our molecular analyses. In particular, we performed a non-standardized survey in the three areas of the island where the possible occurrence of this species was reported by local communities (see Figure 1 and Table 1 for details on sampling stations). The waterbodies of these sampling areas are characterized by slow flowing, irregular streams and temporary natural or artificial ponds, mainly with muddy and poorly vegetated riverbeds. Natural streams are often connected one another by drainage canals, which greatly facilitate crayfish spreading. Live crayfish, collected using fish traps, were delivered to the laboratory to be sacrificed by an anaesthetic overdose, and then stored at −20 °C until the DNA extraction.

### 2.2. Diagnostic Molecular Analysis

Total genomic DNA was isolated from a portion of muscle tissue using the Macherey-Nagel Nucleo Spin Tissue Kit (MACHEREY-NAGEL GmbH & Co. KG, Düren, Germany) following the supplier’s instructions. DNA solutions were quantified using the Nanodrop™ Lite Spectrophotometer (by Thermo Scientific; Waltham, MA, USA), which showed an average yield of approximately 30 ng/μL.

A fragment of the subunit I of the mitochondrial Cytochrome c Oxidase gene (COI) was amplified by standard PCR using universal primers [52]. Reactions were carried out in a total volume of 25 μL. On average, 10 ng of total genomic DNA were combined with 0.6 μM of each primer and one pellet of PuReTaq Ready-To-Go PCR beads (GE Healthcare, Wauwatosa, WI, USA) containing stabilizers, bovine serum albumin (BSA), deoxynucleotide triphosphates, 2.5 units of PuReTaq DNA polymerase, and reaction buffer. When a bead was reconstituted to a 25 μL final volume, the concentration of each dNTP and MgCl_2_ was set at 200 μM and 1.5 mM, respectively. PCRs were performed in a GeneAmp PCR System 9700 Thermal Cycler (Applied Biosystems, Waltham, MA, USA), programmed as follows: 1 cycle of 4 min at 94 °C, 35 cycles of 30 s at 94 °C, 30 s at 48 °C, and 30 s at 72 °C. At the end, a post-treatment of 10 min at 72 °C and a final cooling at 4 °C were carried out. Both positive (high-quality DNA samples from the congeneric *P. clarkii*) and negative controls were used to test the effectiveness of the PCR protocols, and the absence of possible contaminations. Electrophoresis was carried out on 2% agarose gels, prepared using 1× TAE buffer (Tris-Acetate-EDTA, pH 8.3) and stained with Gel Red Nucleic Acid Stain (Biotium Inc., Fremont, CA, USA). PCR products were purified by ExoSAP-IT (USB Corporation, Cleveland, OH, USA) and sequenced for forward and reverse strands (by means of the same primers used for PCR), using an external sequencing core service (Macrogen, Europe, Amsterdam, The Netherlands).

### 2.3. Phylogenetic and Phylogeographic Analyses

All the sequences obtained from crayfish adults and eggs were identified as belonging to the species *Procambarus virginalis* through BLAST analysis implemented in the GenBank nucleotide database (www.ncbi.nlm.nih.gov (accessed on 21 July 2020)) that showed a 100% identity.

Sequences were aligned using the package Clustal Omega [53] (available at https://www.ebi.ac.uk/Tools/msa/clustalo/ (accessed on 4 May 2021)) after a manual checking and editing by means of Unipro UGENE v.35 (by the Unipro Center for Information Technologies, Novosibirsk, Russia) [54].

COI sequences of the marbled crayfish collected in Sardinia were aligned with the sequences belonging to *P. virginalis* and *P. fallax* from other localities (Belgium, Czech Republic, Germany, Italy, Sweden, Japan, and Florida—USA) so far available on GenBank (last update 5 April 2021) (see Table 1 for details). As outgroups, three sequences belonging to the species *P. clarkii* from two different localities were chosen: two from Sardinia (obtained in the present study) (Genbank accession numbers: MZ099652, MZ099653), and one from Alabama (USA) (Genbank accession number: KX417114).

Levels of genetic variation among sequences were assessed estimating the number of polymorphic sites (S), number of haplotypes (H), nucleotide diversity (π), and haplotype diversity (h), using the software package DnaSP 6.12.03 (by Universitat de Barcelona, Barcelona, Spain) [55].

To assess the taxonomic status of crayfish collected in Sardinia, the nucleotide divergence threshold (NDT) method of species delimitation [56] was also performed. The NDT method is based on genetic distances and does not consider the phylogenetic relationships within the dataset. It works on sequences to rank taxa into taxonomic entities applying the fixed threshold of 2% given by Hebert et al. [56] for DNA barcodes, using the pairwise Kimura (1980) two-parameter model (K2P) to compute the matrix of genetic distances [57]. The analysis was performed by means of a script [58] written in the R statistical environment (available at https://cran.r-project.org/ (accessed on 5 May 2021)).

The probabilistic model of sequence evolution that better fit the sequence data was detected using the software JmodelTest 2.1.7 [59]. Based on the best-fitting model, a Bayesian phylogenetic species tree was obtained using the software MrBayes 3.2.7 [60] setting as model parameters: NST = 6, rates = invgamma, ngammacat = 4. Two independent runs, each consisting of four Metropolis-Coupled MCMC chains (one cold and three heated chains), were run simultaneously for 5,000,000 generations, sampling trees every 1000 generations. The first 25% of the 10,000 sampled trees was discarded as burn-in. To assess the convergence of chains, we checked that the Average Standard Deviation of Split Frequencies (ASDSF) approached 0 [60], and the Potential Scale Reduction Factor (PSRF) was around 1 [61], following Scarpa et al. [62]. The phylogenetic tree was visualized and edited using FigTree 1.4.0 (available at http://tree.bio.ed.ac.uk/software/figtree/).

To distinguish genetic clusters, a Principal Coordinate Analysis (PCoA) was also performed on a K2P pairwise genetic distance matrix using *GenAlEX* 6.5 [63]. Thevariation rate among sites was modelled with a gamma distribution and all ambiguous positions were removed for each sequence pair.

A median-joining network [64] was constructed using the software package Network 10.0.0.0 (www.fluxus-engineering.com) to infer the genetic relationships among haplotypes and to detect the occurrence (if any) of discrete genetic clusters. The transitions and transversions were equally weighted. Due to the lack of knowledge on the possible occurrence of retromutation events, the same weight (10) was assigned to each observed polymorphism.

## 3. Results

In the present study, 47 COI sequences of 617 bp length were obtained for *Procambarus virginalis* and deposited in GenBank (see Table 1 for accession numbers). Among the sequences belonging to Sardinian *P. virginalis*, a very low level of genetic divergence was found, with three polymorphic sites resulting in four haplotypes (see Table 2 for details on genetic divergence estimates). A high percentage (93.62%) of Sardinian *P. virginalis* share the same haplotype, while 6.38% of the Sardinian individuals showed a private lineage.

The genetic analysis performed on the dataset including all the Sardinian COI sequences obtained in the present study, and those belonging to *P. virginalis* and *P. fallax* deposited in GenBank to date, evidenced 14 polymorphic sites and 9 haplotypes resulting in a low level of genetic variability (see Table 2 for details on genetic divergence estimates) for *P. virginalis* and *P. fallax*. In particular, three haplotypes were found in Sardinian individuals. The NDT species delimitation method ranked all the sequences belonging to *P. virginalis* (also including the Sardinian sequences) and *P. fallax*, into a unique taxonomic entity.

**Figure 2:**
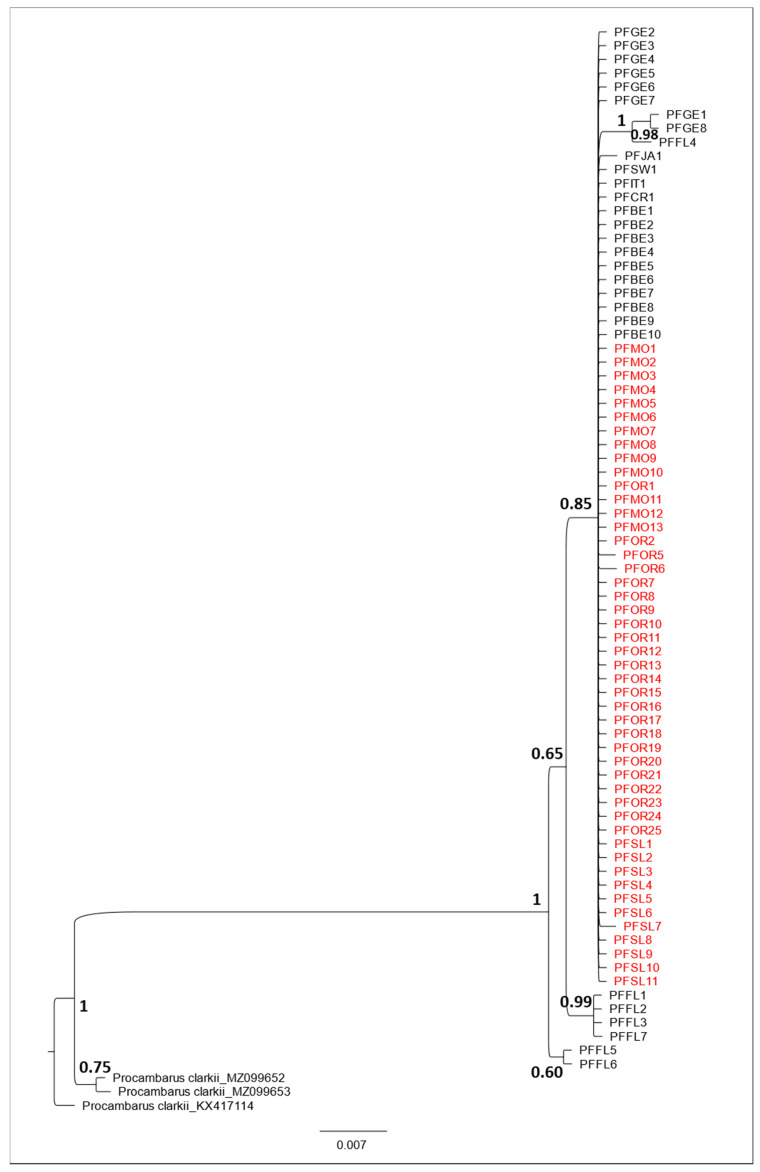
Bayesian phylogenetic tree showing the relationships among *Procambarus virginalis* from Sardinia (indicated with a red font) and marble crayfish from all over the world. Values of support at each node are expressed in Posterior Probabilities. The samples codes are as reported in Table 1.

Accordingly, in the phylogenetic tree (Figure 2), all the sequences belonging to *P. virginalis* and *P. fallax* from Sardinia, other European countries, and Japan, were included in a well-supported monophyletic clade, with an internal sub-cluster that included a sequence of *P. fallax* from Florida (USA) and two sequences of *P. fallax* isolated in Germany. The other sequences belonging to *P. fallax* from Florida were set outside the main clade.

**Figure 3:**
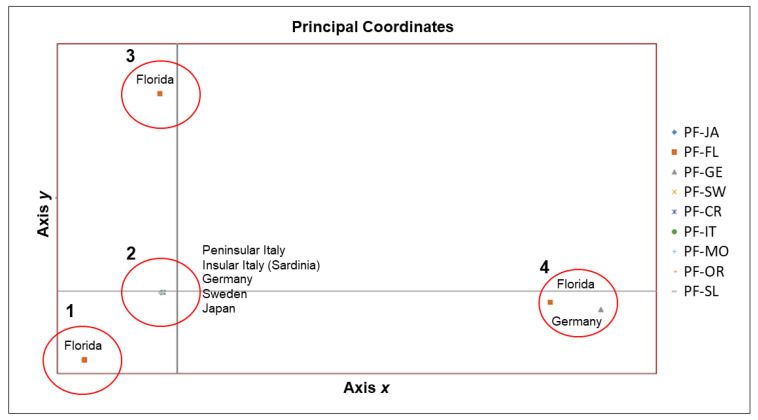
The plot of the Principal Coordinate Analysis (PCoA) evidences the genetic relationships among sequences (and sample sites) based on a matrix of genetic distances. The codes of sequences indicated on the right are as reported in Table 1.

The PCoA plot (Figure 3) showed the occurrence of four groups of sequences (groups 1–4). A genetic similarity was found along the *x*-axis between the group 2—including all the Sardinian sequences and almost all the sequences of *P. virginalis* and *P. fallax* from European countries and Japan—and the groups 1 and 3 which included sequences of *P. fallax* isolated in Florida (see Appendix A Table A1 for details). A further divergent group (group 4) of three sequences, already evidenced in the phylogenetic tree (Figure 2), was separated along the *x*-axis, and included two sequences of *P. fallax* isolated in Germany and one sequence of *P. fallax* isolated in Florida.

**Figure 4:**
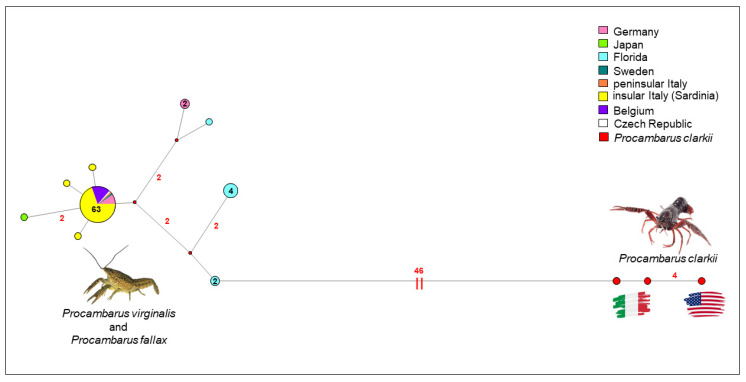
The median-joining network shows the relationships among COI gene haplotypes. The small red spots on the nodes show median vectors representing the hypothetical connecting sequences that were calculated using the maximum parsimony method. The numbers of mutations between sequences that are greater than 1 are reported on network branches.

The network analysis (Figure 4) showed a typical star-like shape, with the occurrence of a common haplotype shared by 93.75% and 25.00% of individuals belonging to *P. virginalis* and *P. fallax* (all collected in Germany and Sweden), respectively. Four sequences of *P. virginalis* diverged from the common haplotype by one- or two-point mutations; three of them were isolated in Sardinia (two from Arborea and one from Sanluri) and one in Japan. Further sequences belonging to *P. fallax* collected in Florida and Germany diverged by 3- to 4-point mutations.

## 4. Discussion

This paper reports the first record of *Procambarus virginalis* in Sardinia, supported by molecular identification. This study also represents the first insight into the genetic variability of the first Italian population of *P. virginalis*, as only one individual from Tuscany [10] and three from Veneto [65] are known from Italy to date. New inferences are also provided on the global genetic variation of this species.

Overall, the Sardinian population of the marble crayfish is characterized by a very low level of genetic variability, with a high percentage of specimens sharing the same haplotype, apart from three individuals which show new, slightly divergent haplotypes. Two possible scenarios may be invoked to explain this remarkable finding: (1) the parthenogenetic marble crayfish was introduced in Sardinia with a highly variable group of individuals, and further studies, involving a greater number of specimens from areas outside Sardinia, are needed to check for the occurrence of these never reported haplotypes in the mainland; or (2) based on the current knowledge, we cannot rule out that the three private haplotypes found in Sardinia might stem as the result of strong selective pressures acting on the population of this recently introduced invasive species.

Indeed, all the sequences belonging to *P. virginalis* from European countries are identical, with only few exceptions. The most common haplotype of *P. virginalis* also occurs in *P. fallax* from Germany and Sweden, which may represent individuals directly imported from the USA. Interestingly, no one of the COI sequences isolated in *P. fallax* from the USA correspond to the most common lineage found in *P. virginalis* from Europe and Japan. The lack of the most common, worldwide spread haplotype of *P. virginalis* among the sequences of *P. fallax* from the USA might reflect their low number so far available on GenBank. However, this pattern may also depend on the genetic drift resulting from the small number of founders selected in the USA to be imported in Europe for the pet trade. In this latter case, only a small and poorly representative portion of the source population genetic variability might have been transferred to the European populations, thus resulting in the lack of shared haplotypes between the two continents.

Furthermore, when adaptive evolution takes place, the molecular lineages of introduced populations can be rare, or not expressed in the source population but become common and distinctive among individuals in the new colonized habitats as a consequence of founder events and natural selection [66]. Although representing an uncommon trend, selective pressure on mtDNA promoted by new environmental conditions was already reported for the highly invasive Lessepsian migrant *Fistularia commersonii* [67], whose recently established Mediterranean population does not include any mitochondrial lineages present among individuals of the source population. For this reason, in the case of *P. virginalis,* mitochondrial variants, arrived in Europe from the USA, may have undergone a selective sweep which might have favoured the spread in European populations of rare alleles that likely allow a better adaptation of the species to new environmental conditions.

## 5. Conclusions

Although there is no evidence of the presence of native crayfish in Sardinia, the autochthonous nature of *Austrapotamobius pallipes*, included in Annexes II and V of the European Union Habitats Directive 2000 (92/43/EEC), is still controversial. Nonetheless, there are endemic species of amphibians that may suffer due to the presence of this new invasive alien species in their fresh waters. For instance, Souty-Grosset et al. [68] and Oficialdegui et al. [69] showed that crayfish eat amphibian eggs and tadpoles. Additionally, competition for food could also indirectly reduce amphibian populations, as the grazing activity that crayfish exert on periphyton may lead periphyton-associated invertebrates to disappear [70]. Lastly, there is evidence that crayfish could carry the chytrid *Batrachochytrium dendrobatidis* (see Battisti and Scalici [26] and references therein), which may cause diseases in populations of Sardinian endemic amphibians.

Therefore, future studies that investigate the stomach content of these crayfish would be useful to provide a clear idea of their impact on Sardinian native fauna. At the same time, further studies are needed to investigate the presence in the island of the oomycete *Aphanomyces astaci,* which is generally associated to crayfish. Indeed, the presence of *A. astaci* in other crustaceans, such as *Eriocheir sinensis* [71,72], suggests the occurrence of spill over events.

## Figures and Tables

**Table 1:** The table reports data on the sampling collection. Sampling sites for each geographic area are indicated for the individuals of *Procambarus virginalis* collected in Sardinia during the present study. Details are also provided for the GenBank sequences of *P. virginalis* and *P. fallax* from all over the world used for the analyses.

Sample ID	Sampling Site	Species	GB #	Coordinates		Habitat	Sampling Date
				Long	Lat		
PFMO1	Italy (Sardinia)—Morimenta	*Procambarus virginalis*	MZ097549	8,68540286	39,6536863	Creek	May 2019
PFMO2	Italy (Sardinia)—Morimenta	*Procambarus virginalis*	MZ097550	8,68540286	39,6536863	Creek	May 2019
PFMO3	Italy (Sardinia)—Morimenta	*Procambarus virginalis*	MZ097551	8,68540286	39,6536863	Creek	May 2019
PFMO7	Italy (Sardinia)—Morimenta	*Procambarus virginalis*	MZ097555	8,68540286	39,6536863	Creek	May 2019
PFOR1	Italy (Sardinia)—Arborea	*Procambarus virginalis*	MZ097559	8,571233244	39,72729907	Creek	May 2019
PFMO4	Italy (Sardinia)—Morimenta	*Procambarus virginalis*	MZ097552	8,68540286	39,6536863	Creek	June 2019
PFMO5	Italy (Sardinia)—Morimenta	*Procambarus virginalis*	MZ097553	8,68540286	39,6536863	Creek	June 2019
PFMO6	Italy (Sardinia)—Morimenta	*Procambarus virginalis*	MZ097554	8,68540286	39,6536863	Creek	June 2019
PFMO8	Italy (Sardinia)—Morimenta	*Procambarus virginalis*	MZ097556	8,68540286	39,6536863	Creek	June 2019
PFMO9	Italy (Sardinia)—Morimenta	*Procambarus virginalis*	MZ097557	8,68540286	39,6536863	Creek	June 2019
PFMO10	Italy (Sardinia)—Morimenta	*Procambarus virginalis*	MZ097558	8,68540286	39,6536863	Creek	June 2019
PFMO11	Italy (Sardinia)—Morimenta	*Procambarus virginalis*	MZ097560	8,68540286	39,6536863	Creek	June 2019
PFMO12	Italy (Sardinia)—Morimenta	*Procambarus virginalis*	MZ097561	8,68540286	39,6536863	Creek	June 2019
PFMO13	Italy (Sardinia)—Morimenta	*Procambarus virginalis*	MZ097562	8,68540286	39,6536863	Creek	June 2019
PFOR2	Italy (Sardinia)—Arborea	*Procambarus virginalis*	MZ097563	8,571233244	39,72729907	Creek	November 2019
PFOR5	Italy (Sardinia)—Arborea	*Procambarus virginalis*	MZ097564	8,571233244	39,72729907	Creek	November 2019
PFOR6	Italy (Sardinia)—Arborea	*Procambarus virginalis*	MZ097565	8,571233244	39,72729907	Creek	November 2019
PFOR7	Italy (Sardinia)—Arborea	*Procambarus virginalis*	MZ097566	8,571233244	39,72729907	Creek	November 2019
PFOR8	Italy (Sardinia)—Arborea	*Procambarus virginalis*	MZ097567	8,571233244	39,72729907	Creek	November 2019
PFOR9	Italy (Sardinia)—Arborea	*Procambarus virginalis*	MZ097568	8,571233244	39,72729907	Creek	November 2019
PFOR10	Italy (Sardinia)—Arborea	*Procambarus virginalis*	MZ097569	8,571233244	39,72729907	Creek	November 2019
PFOR11	Italy (Sardinia)—Arborea	*Procambarus virginalis*	MZ097570	8,621508481	39,82035494	Creek	November 2019
PFOR12	Italy (Sardinia)—Arborea	*Procambarus virginalis*	MZ097571	8,621508481	39,82035494	Creek	November 2019
PFOR13	Italy (Sardinia)—Arborea	*Procambarus virginalis*	MZ097572	8,621508481	39,82035494	Creek	November 2019
PFOR14	Italy (Sardinia)—Arborea	*Procambarus virginalis*	MZ097573	8,621508481	39,82035494	Creek	November 2019
PFOR15	Italy (Sardinia)—Arborea	*Procambarus virginalis*	MZ097574	8,621508481	39,82035494	Creek	November 2019
PFOR16	Italy (Sardinia)—Arborea	*Procambarus virginalis*	MZ097575	8,578457118	39,82507761	Creek	November 2019
PFOR17	Italy (Sardinia)—Arborea	*Procambarus virginalis*	MZ097576	8,578457118	39,82507761	Creek	November 2019
PFOR18	Italy (Sardinia)—Arborea	*Procambarus virginalis*	MZ097577	8,549284943	39,74396601	Creek	November 2019
PFOR19	Italy (Sardinia)—Arborea	*Procambarus virginalis*	MZ097578	8,60873508	39,8203551	Creek	November 2019
PFOR20	Italy (Sardinia)—Arborea	*Procambarus virginalis*	MZ097579	8,60873508	39,8203551	Creek	November 2019
PFOR21	Italy (Sardinia)—Arborea	*Procambarus virginalis*	MZ097580	8,60873508	39,8203551	Creek	November 2019
PFOR22	Italy (Sardinia)—Arborea	*Procambarus virginalis*	MZ097581	8,60873508	39,8203551	Creek	November 2019
PFOR23	Italy (Sardinia)—Arborea	*Procambarus virginalis*	MZ097582	8,60873508	39,8203551	Creek	November 2019
PFOR24	Italy (Sardinia)—Arborea	*Procambarus virginalis*	MZ097583	8,60873508	39,8203551	Creek	November 2019
PFOR25	Italy (Sardinia)—Arborea	*Procambarus virginalis*	MZ097584	8,60873508	39,8203551	Creek	November 2019
PFSL1	Italy (Sardinia)—Sanluri	*Procambarus virginalis*	MZ097585	8,842628592	39,51785049	Creek	December 2019
PFSL2	Italy (Sardinia)—Sanluri	*Procambarus virginalis*	MZ097586	8,842628592	39,51785049	Creek	December 2019
PFSL3	Italy (Sardinia)—Sanluri	*Procambarus virginalis*	MZ097587	8,842628592	39,51785049	Creek	December 2019
PFSL4	Italy (Sardinia)—Sanluri	*Procambarus virginalis*	MZ097588	8,842628592	39,51785049	Creek	December 2019
PFSL5	Italy (Sardinia)—Sanluri	*Procambarus virginalis*	MZ097589	8,842628592	39,51785049	Creek	December 2019
PFSL6	Italy (Sardinia)—Sanluri	*Procambarus virginalis*	MZ097590	8,844295005	39,51785048	Creek	December 2019
PFSL7	Italy (Sardinia)—Sanluri	*Procambarus virginalis*	MZ097591	8,844295005	39,51785048	Creek	December 2019
PFSL8	Italy (Sardinia)—Sanluri	*Procambarus virginalis*	MZ097592	8,844295005	39,51785048	Creek	December 2019
PFSL9	Italy (Sardinia)—Sanluri	*Procambarus virginalis*	MZ097593	8,844295005	39,51785048	Creek	December 2019
PFSL10	Italy (Sardinia)—Sanluri	*Procambarus virginalis*	MZ097594	8,844295005	39,51785048	Creek	December 2019
PFSL11	Italy (Sardinia)—Sanluri	*Procambarus virginalis*	MZ097595	8,844295005	39,51785048	Creek	December 2019
PFBE8	Belgium	*Procambarus virginalis*	LR884227	-	-	Pond	May 2020
PFBE9	Belgium	*Procambarus virginalis*	LR884226	-	-	Pond	May 2020
PFBE10	Belgium	*Procambarus virginalis*	LR884225	-	-	Moat	May 2020
PFBE5	Belgium	*Procambarus virginalis*	LR884230	-	-	Pond	June 2020
PFBE6	Belgium	*Procambarus virginalis*	LR884229	-	-	Pond	June 2020
PFBE7	Belgium	*Procambarus virginalis*	LR884228	-	-	Basin	June 2020
PFBE1	Belgium	*Procambarus virginalis*	LR884234	-	-	Moat	July 2020
PFBE2	Belgium	*Procambarus virginalis*	LR884233	-	-	Moat	July 2020
PFBE3	Belgium	*Procambarus virginalis*	LR884232	-	-	Pond	July 2020
PFBE4	Belgium	*Procambarus virginalis*	LR884231	-	-	Pond	July 2020
PFCR1	Czech Republic	*Procambarus virginalis*	MK439899	-	-	-	-
PFIT1	Italy (Veneto)	*Procambarus virginalis*	KJ690261	-	-	Channel	April 2009
PFJA1	Japan	*Procambarus virginalis*	LC228303	-	-	-	November 2016
PFGE2	Germany	*Procambarus virginalis*	HM358011	-	-	Stream	October 2009
PFGE5	Germany	*Procambarus virginalis*	KC107813	-	-	-	-
PFGE6	Germany	*Procambarus virginalis*	KT074364	-	-	-	-
PFGE4	Germany	*Procambarus virginalis*	HM358010	-	-	Stream	October 2009
PFGE1	Germany	*Procambarus fallax*	HM358012	-	-	-	October 2009
PFGE3	Germany	*Procambarus fallax*	JF438007	-	-	Lake	-
PFGE8	Germany	*Procambarus fallax*	KT074365	-	-	-	-
PFGE7	Germany	*Procambarus fallax*	NC_020021	-	-	-	-
PFFL1	Florida—USA	*Procambarus fallax*	HQ171459	-	-	-	-
PFFL2	Florida—USA	*Procambarus fallax*	HQ171458	-	-	-	-
PFFL3	Florida—USA	*Procambarus fallax*	HQ171457	-	-	-	-
PFFL4	Florida—USA	*Procambarus fallax*	HQ171456	-	-	-	-
PFFL5	Florida—USA	*Procambarus fallax*	HQ171455	-	-	-	-
PFFL6	Florida—USA	*Procambarus fallax*	HQ171453	-	-	-	-
PFFL7	Florida—USA	*Procambarus fallax*	HQ171454	-	-	-	-
PFSW1	Sweden	*Procambarus fallax*	KF033123	-	-	River	December 2012

# stands for accession number.

**Table 2:** Genetic divergence estimates for *Procambarus virginalis* and *Procambarus fallax* based on COI gene sequences.

	N	bp	*S*	*H*	*hd*	*π*
Sardinia	47	617	3	4	0.125 ± 0.065	0.00021
Whole dataset	76	617	14	9	0.312 ± 0.069	0.00178

## Data Availability

Sequences obtained in the present study for the mitochondrial Cytochrome c Oxidase subunit I gene isolated in Sardinian crayfish were deposited in the GenBank database under the accession numbers MZ097549-95, MZ099652-53.

## Appendix A

**Table A1:** Principal Coordinate Analysis (PCoA) results. The table shows the specific composition of the four groups of sequences.

**Group 1**		
**NORTH AMERICA**		
**Sample ID**	**Country**	**Species**
PFFL1	Florida—USA	*Procambarus fallax*
PFFL2	Florida—USA	*Procambarus fallax*
PFFL3	Florida—USA	*Procambarus fallax*
PFFL7	Florida—USA	*Procambarus fallax*
**Group 2**		
**EUROPE**		
**Sample ID**	**Country**	**Species**
PFGE2	Germany	*Procambarus fallax*
PFGE3	Germany	*Procambarus fallax*
PFGE4	Germany	*Procambarus virginalis*
PFGE5	Germany	*Procambarus virginalis*
PFGE6	Germany	*Procambarus virginalis*
PFGE7	Germany	*Procambarus virginalis*
PFCR1	Czech Republic	*Procambarus virginalis*
PFSW1	Sweden	*Procambarus virginalis*
PFBE1	Belgium	*Procambarus virginalis*
PFBE2	Belgium	*Procambarus virginalis*
PFBE3	Belgium	*Procambarus virginalis*
PFBE4	Belgium	*Procambarus virginalis*
PFBE5	Belgium	*Procambarus virginalis*
PFBE6	Belgium	*Procambarus virginalis*
PFBE7	Belgium	*Procambarus virginalis*
PFBE8	Belgium	*Procambarus virginalis*
PFBE9	Belgium	*Procambarus virginalis*
PFBE10	Belgium	*Procambarus virginalis*
PFIT1	Peninsular Italy—Veneto	*Procambarus virginalis*
PFMO1	Insular Italy—Sardinia	*Procambarus virginalis*
PFMO2	Insular Italy—Sardinia	*Procambarus virginalis*
PFMO3	Insular Italy—Sardinia	*Procambarus virginalis*
PFMO4	Insular Italy—Sardinia	*Procambarus virginalis*
PFMO5	Insular Italy—Sardinia	*Procambarus virginalis*
PFMO6	Insular Italy—Sardinia	*Procambarus virginalis*
PFMO7	Insular Italy—Sardinia	*Procambarus virginalis*
PFMO8	Insular Italy—Sardinia	*Procambarus virginalis*
PFMO9	Insular Italy—Sardinia	*Procambarus virginalis*
PFMO10	Insular Italy—Sardinia	*Procambarus virginalis*
PFMO11	Insular Italy—Sardinia	*Procambarus virginalis*
PFMO12	Insular Italy—Sardinia	*Procambarus virginalis*
PFMO13	Insular Italy—Sardinia	*Procambarus virginalis*
PFOR1	Insular Italy—Sardinia	*Procambarus virginalis*
PFOR2	Insular Italy—Sardinia	*Procambarus virginalis*
PFOR5	Insular Italy—Sardinia	*Procambarus virginalis*
PFOR6	Insular Italy—Sardinia	*Procambarus virginalis*
PFOR7	Insular Italy—Sardinia	*Procambarus virginalis*
PFOR8	Insular Italy—Sardinia	*Procambarus virginalis*
PFOR9	Insular Italy—Sardinia	*Procambarus virginalis*
PFOR10	Insular Italy—Sardinia	*Procambarus virginalis*
PFOR11	Insular Italy—Sardinia	*Procambarus virginalis*
PFOR12	Insular Italy—Sardinia	*Procambarus virginalis*
PFOR13	Insular Italy—Sardinia	*Procambarus virginalis*
PFOR14	Insular Italy—Sardinia	*Procambarus virginalis*
PFOR15	Insular Italy—Sardinia	*Procambarus virginalis*
PFOR16	Insular Italy—Sardinia	*Procambarus virginalis*
PFOR17	Insular Italy—Sardinia	*Procambarus virginalis*
PFOR18	Insular Italy—Sardinia	*Procambarus virginalis*
PFOR19	Insular Italy—Sardinia	*Procambarus virginalis*
PFOR20	Insular Italy—Sardinia	*Procambarus virginalis*
PFOR21	Insular Italy—Sardinia	*Procambarus virginalis*
PFOR22	Insular Italy—Sardinia	*Procambarus virginalis*
PFOR23	Insular Italy—Sardinia	*Procambarus virginalis*
PFOR24	Insular Italy—Sardinia	*Procambarus virginalis*
PFOR25	Insular Italy—Sardinia	*Procambarus virginalis*
PFSL1	Insular Italy—Sardinia	*Procambarus virginalis*
PFSL2	Insular Italy—Sardinia	*Procambarus virginalis*
PFSL3	Insular Italy—Sardinia	*Procambarus virginalis*
PFSL4	Insular Italy—Sardinia	*Procambarus virginalis*
PFSL5	Insular Italy—Sardinia	*Procambarus virginalis*
PFSL6	Insular Italy—Sardinia	*Procambarus virginalis*
PFSL7	Insular Italy—Sardinia	*Procambarus virginalis*
PFSL8	Insular Italy—Sardinia	*Procambarus virginalis*
PFSL9	Insular Italy—Sardinia	*Procambarus virginalis*
PFSL10	Insular Italy—Sardinia	*Procambarus virginalis*
PFSL11	Insular Italy—Sardinia	*Procambarus virginalis*
**ASIA**		
**Sample ID**	**Country**	**Species**
PFJA1	Japan	*Procambarus virginalis*
**Group 3**		
**NORTH AMERICA**		
**Sample ID**	**Country**	**Species**
PFFL5	Florida—USA	*Procambarus fallax*
PFFL6	Florida—USA	*Procambarus fallax*
**Group 4**		
**NORTH AMERICA**		
**Sample ID**	**Country**	**Species**
PFFL4	Florida—USA	*Procambarus fallax*
**EUROPE**		
**Sample ID**	**Country**	**Species**
PFGE1	Germany	*Procambarus fallax*
PFGE8	Germany	*Procambarus fallax*
